# Genetic Variations in Key MicroRNAs are Associated With the Survival of Nonsmall Cell Lung Cancer

**DOI:** 10.1097/MD.0000000000002084

**Published:** 2015-10-30

**Authors:** Shuangshuang Wu, Wei Shen, Yun Pan, Meng Zhu, Kaipeng Xie, Liguo Geng, Yuzhuo Wang, Yan Liang, Jiali Xu, Songyu Cao, Wei Xu, Bo Chen, Zhibin Hu, Hongxia Ma, Jianqing Wu, Hongbing Shen

**Affiliations:** From the Department of Geriatrics (SW, YL, WX, BC, JW); Editorial Department of Journal of Clinical Dermatology (YP); Department of Oncology, The First Affiliated Hospital of Nanjing Medical University (JX); Department of Epidemiology and Biostatistics, School of Public Health, Nanjing Medical University (WS, MZ, KX, LG, YW, ZH, HM); Jiangsu Key Lab of Cancer Biomarkers, Prevention and Treatment, Collaborative Innovation Center for Cancer Personalized Medicine, Nanjing Medical University (WS, MZ, KX, LG, YW, ZH, HM, HS); and State-Level Model Center of Experimental Teaching, Department of Hygienic Analysis and Detection, School of Public Health, Nanjing Medical University, Nanjing, Jiangsu, P.R. China (SC).

## Abstract

MicroRNAs (miRNAs) are a class of small, noncoding RNA molecules involved in carcinogenesis. It has been identified that genetic variations in miRNAs contribute to cancer risk, prognosis, and survival. In the present study, we investigated whether single nucleotide polymorphisms (SNPs) of several key miRNAs (*miR-184*, *miR-218*, and *miR-124*) were associated with the prognosis of nonsmall cell lung cancer (NSCLC) in a clinical cohort study including 1001 cases. Cox proportional hazards regression models were used to estimate the hazard ratios (HRs) and their 95% confidence intervals (CIs). We found that 5 SNPs were associated with NSCLC survival (rs919968, rs3775815, rs4867902, and rs6122390 in an additive model: adjusted HR = 1.15, 95% CI = 1.02–1.29; adjusted HR = 0.78, 95% CI = 0.67–0.91, adjusted HR = 1.24, 95% CI = 1.09–1.41; adjusted HR = 1.21, 95% CI = 1.07–1.36, respectively; rs298206 in a dominant model: HR = 1.25, 95% CI = 1.05–1.49). Even after the Bonferroni correction, 3 SNPs remained significant (adjusted *P* = 0.010, 0.010, and 0.032 for rs3775815, rs4867902, and rs6122390, respectively). Additionally, the combined analysis of these 5 SNPs showed a significant locus-dosage effect between number of unfavorable alleles (rs919968-A, rs3775815-C, rs4867902-G, rs6122390-A, and rs298206-T) and death risk of NSCLC (*P* for trend < 0.001). A statistically significant multiplicative interaction was found between the genotypes of rs4867902 and surgical operation status (*P*_int_ = 0.013). These findings indicated that genetic variations in miRNAs (*miR-184*, *miR-218*, and *miR-124*) might be prognostic markers for NSCLC patients.

## INTRODUCTION

Lung cancer, mainly nonsmall cell lung cancer (NSCLC), contributes to one of the primary cancer deaths throughout the world, with a 5-year survival rate of less than 15%.^1^ The tumor size and staging have been widely used to predict prognosis and response to therapy. However, recent studies indicate that the combination of specific prognostic biomarkers and traditional clinical prognostic factors can add value for prognosis and individualized treatment of NSCLC.^2^

MicroRNAs (MiRNAs) are small highly conserved noncoding RNAs which usually regulate the gene expression after transcription through binding to target mRNAs.^3^ Published data indicated that many miRNAs were upregulated or downregulated in diverse carcinomas and their aberrant expression levels were associated with tumorigenesis or the prognosis of various human cancers.^4–6^ Recently, several publications have reported that differential expression of several important miRNAs, such as *miR-184*, the members of *miR-218* and *miR-124* family, are essential to the survival of numerous types of cancer including lung cancer.^7–17^ For example, some studies have shown that upregulation of *miR-184* is associated with survival of several types of cancer^7,8^; decreased *miR-218* expression has been detected in various types of cancer and linked with the worse survival of lung and colon tumors^9–13^; and deregulated *miR-124* is a prognostic factor in patients with NSCLC.^13,17^

MiRNAs-related single nucleotide polymorphisms (SNPs) could change miRNA-mediated regulation and thus affect cancer prognosis and survival.^18^ To date, no study has investigated the relationship between *hsa-miR-184*, *hsa-miR-218* family (*hsa-miR-218-1*, *hsa-miR-218-2*) and *hsa-miR-124* family (*hsa-miR-124-1*, *hsa-miR-124-2*, *hsa-miR-124-3*) polymorphisms and survival of NSCLC. Therefore, in this study, we selected all common (minor allele frequency (MAF) ≥0.1) in Chinese population and potentially functional SNPs from the 6 miRNAs (*miR-184*, *miR-218–1*, *miR-218-2*, *miR-124-1*, *miR-124-2*, and *miR-124-3*) and their surrounding regions to estimate the associations with NSCLC survival.

## METHODS

### Ethics Statement

Our study was authorized by the Institutional Review Board of Nanjing Medical University. All the participants have signed the informed consent before participating in the study.

### Study Population

Since July 2003, a total of 1341 NSCLC patients have been enlisted from the Cancer Hospital of Jiangsu Province, and the First Affiliated Hospital of Nanjing Medical University, Nanjing, China. All patients were unrelated ethnic Han Chinese population (CHB).^19,20^ They were new NSCLC cases and were histopathologically or cytologically confirmed by at least 2 local pathologists. The patients had no history of other cancers or previous chemotherapy or radiotherapy. A structured questionnaire on demographic and exposure information, including age, gender, and tobacco consumption, was conducted by qualified investigators through face-to-face interviews with the patients. Nonsmokers were defined as those who smoked less than 1 cigarette per day and less than 1 year over their lifetime; otherwise, they were considered as smokers. In addition, 5-ml fasting venous blood each was collected for genomic DNA extraction. Each patient was followed up every 3 months to collect the information of treatment and progression of the disease. Until the last follow-up of August 2013, 1001 cases (74.6%) had completed demographic and follow-up information and provided adequate DNA sample.

### SNPs Selection and Genotyping

We used HapMap database (phase II + III Feb 09, dbSNP b126) and the Haploview 4.1 software to identify common SNPs in miRNA gene regions, meeting the following criteria: containing the region in and upstream 10 kb of the miRNAs; MAF ≥ 0.1 in CHB population; Hardy–Weinberg equilibrium (HWE) test, *P* > 0.05; call rate ≥90%. Next, we used SNP info Web Server (http://snpinfo.niehs.nih.gov/) to indicate the potential function of SNPs. Linkage disequilibrium (LD) analysis with an r^2^ threshold of 0.80 was also applied to select SNPs. No SNPs were selected from *miR-124-1*. Finally, 11 loci from other 5 miRNAs were finally selected.

Blood samples were collected in EDTA anticoagulant tubes and stored at −80°C until DNA extraction. Following the standard protocols, genomic DNA was obtained through proteinase K digestion and phenol/chloroform extraction. The genotyping and genotype calling was conducted by Illumina Infinium® BeadChip and GenTrain version 1.0 clustering algorithm in GenomeStudio V2011.1 (Illumina, Inc.), respectively. Quality control was performed according to the quality criteria (ie, 1 blank well and 3 paralleled samples) mentioned in our previous studies.^21^ Finally, 10 SNPs were successfully genotyped, whereas rs9784690 (*miR-218-2*) was excluded because of design failure.

### Statistical Analyses

The survival time was obtained by calculating from the time of diagnosis until death or the latest follow-up. Goodness-of-fit χ^2^ test was applied to evaluate HWE. Correlation between the genotype and death risk was estimated by the Kaplan–Meier method and log-rank test. Cox proportional hazard regression analysis were conducted to estimate the crude and adjusted HR and their 95% confidence interval (CI). Adjusted variables were age, gender, smoking status, clinical stage, chemoradiotherapy status, surgery status, and histology. The Chi-square-based *Q* test was used to examine the heterogeneity between subgroups. The possible gene–environment (ie, surgical operation) interactions were also evaluated by the Cox proportional hazard regression models. Analyses were carried out through Statistical Analysis System software (version 9.1.3; SAS Institute, Cary, NC). All tests were 2-sided and statistically significant threshold was *P* < 0.05.

## RESULTS

### Patients’ Characteristics and Clinical Features

Sociodemographic and clinical features of NSCLC patients are displayed in Table 1. For the 1001 patients retained in the study, 48.8 % was older than 62 years old and 69.4% was male. During the follow-up period, 545 patients died from NSCLC and the median survival time (MST) of all the patients was 26.0 months. No significant difference of NSCLC-specific survival was shown between subgroups of age and histology types (*P* = 0.418 and 0.060). Smokers, patients with advanced stage NSCLC and patients received chemotherapy or radiotherapy had significantly shorter MST, whereas females and surgical resection remarkably improve the survival of NSCLC (log-rank *P* < 0.05, Table 1).

**TABLE 1:**
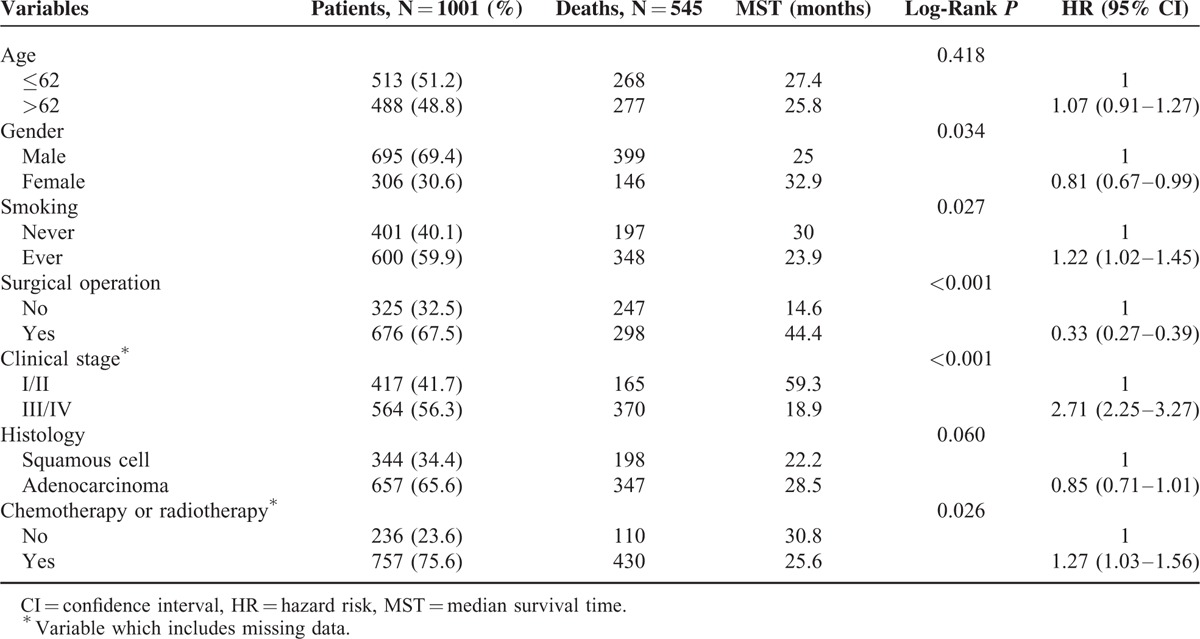
Patients’ Characteristics and Clinical Features

### Effects of Polymorphisms on the Survival of NSCLC

Among the 10 SNPs, 6 SNPs (rs919968, rs3775815, rs4867902, rs298210, rs298206, and rs6122390) had a log-rank *P* under 0.05 in any of the genetic models (Table 2). However, after adjustment for age, gender, smoking status, clinical stage, chemoradiotherapy status, surgery status, and histology, 5 of them remained significant associations with the prognosis of NSCLC. Among the 5 SNPs, 4 SNPs were associated with worse NSCLC survival (additive model: rs919968, adjusted hazard ratio (HR) = 1.15, 95% CIs = 1.02–1.29, *P* = 0.027; rs4867902, adjusted HR = 1.24, 95% CI = 1.09–1.41, *P* = 0.001; and rs6122390, adjusted HR = 1.21, 95% CI = 1.07–1.36, *P* = 0.003; dominant model: rs298206, adjusted HR = 1.25, 95% CI = 1.05–1.49, *P* = 0.012) while rs3775815 was associated with better NSCLC survival (additive model: adjusted HR = 0.78, 95% CI = 0.67–0.91, *P* = 0.001) (Table 3). Additionally, the Bonferroni correction was also used to reduce the chances of obtaining false-positive results and 3 SNPs remained significant (adjusted *P* = 0.010, 0.010, and 0.030 for rs3775815, rs4867902, and rs6122390, respectively).

**TABLE 2:**
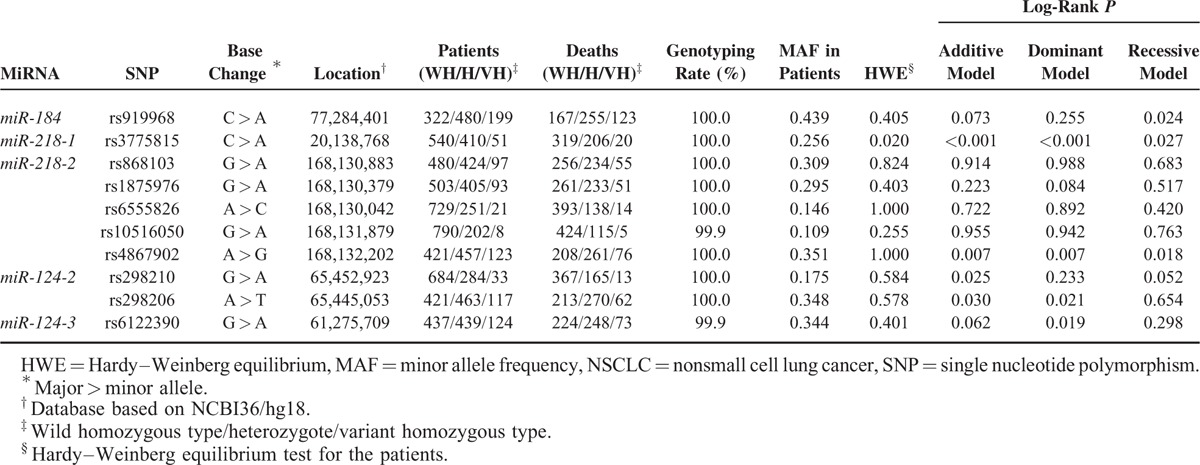
Genotyping Results With NSCLC Patients’ Survival

**TABLE 3:**
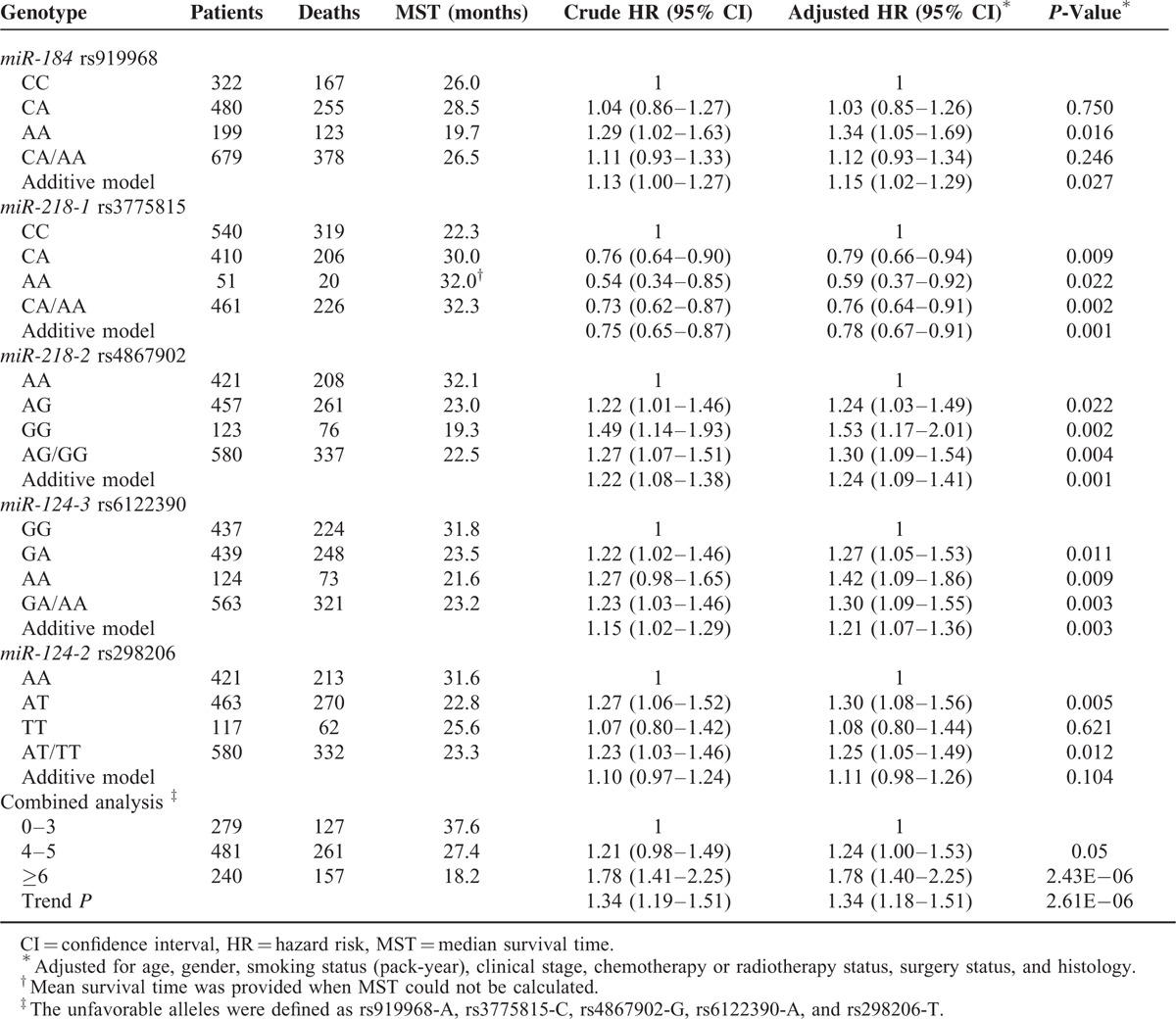
Genotypes of rs919968, rs3775815, rs4867902, rs6122390, rs298206 and Lung Cancer Patients’ Survival

**FIGURE 1:**
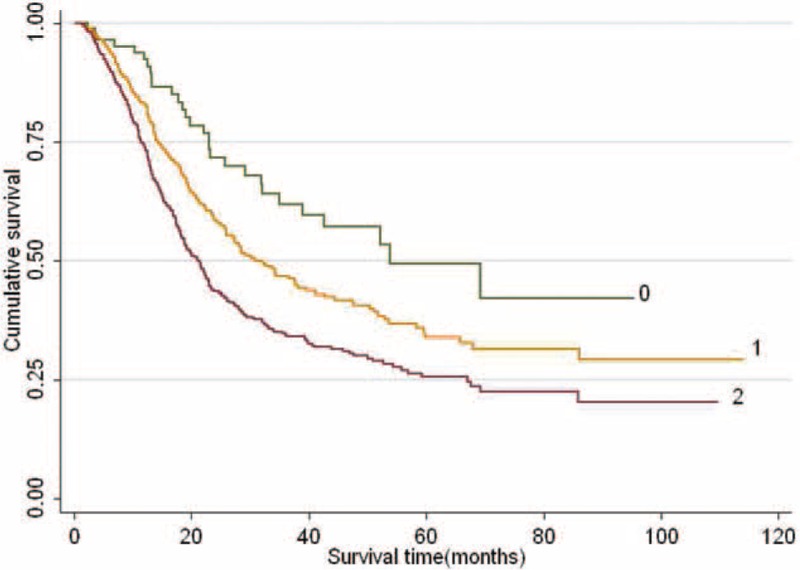
Kaplan–Meier plots of survival for combined effect of the 5 SNPs on NSCLC-specific survival. SNPs: rs919968-A, rs3775815-C, rs4867902-G, rs6122390-A, and rs298206-T; “0” denotes patients carried 0 to 3 unfavorable alleles, “1” means patients carried 4 to 5 unfavorable alleles, “2” means patients carried more than 6 unfavorable alleles.

Additionally, combined effects on NSCLC survival were calculated by adding up the number of unfavorable alleles of the independent SNPs (rs919968-A, rs3775815-C, rs4867902-G, rs6122390-A, and rs298206-T), and a significant locus-response relationship was observed (*P* for trend < 0.001, Table 3). As shown in Table 3, compared to subjects with “0–3” unfavorable alleles (MST = 37.6 months), subjects carrying more unfavorable loci had the shorter MST (MST = 27.4 and18.2 months for “4–5” and “≥6” unfavorable alleles, respectively; log-rank *P* < 0.001; Figure 1).

### Stratification and Interaction Analysis

The effects of the 5 polymorphisms on NSCLC survival were further evaluated by stratification analysis on age, gender, smoking status, surgical operation, stage, histology, and chemoradiotherapy. As shown in Table 4, we found that increased HR of rs4867902 was more evident for patients who did not experience surgery (adjusted HR = 1.51, 95% CI = 1.25–1.82, *P* = 0.008 for heterogeneity test).

**TABLE 4:**
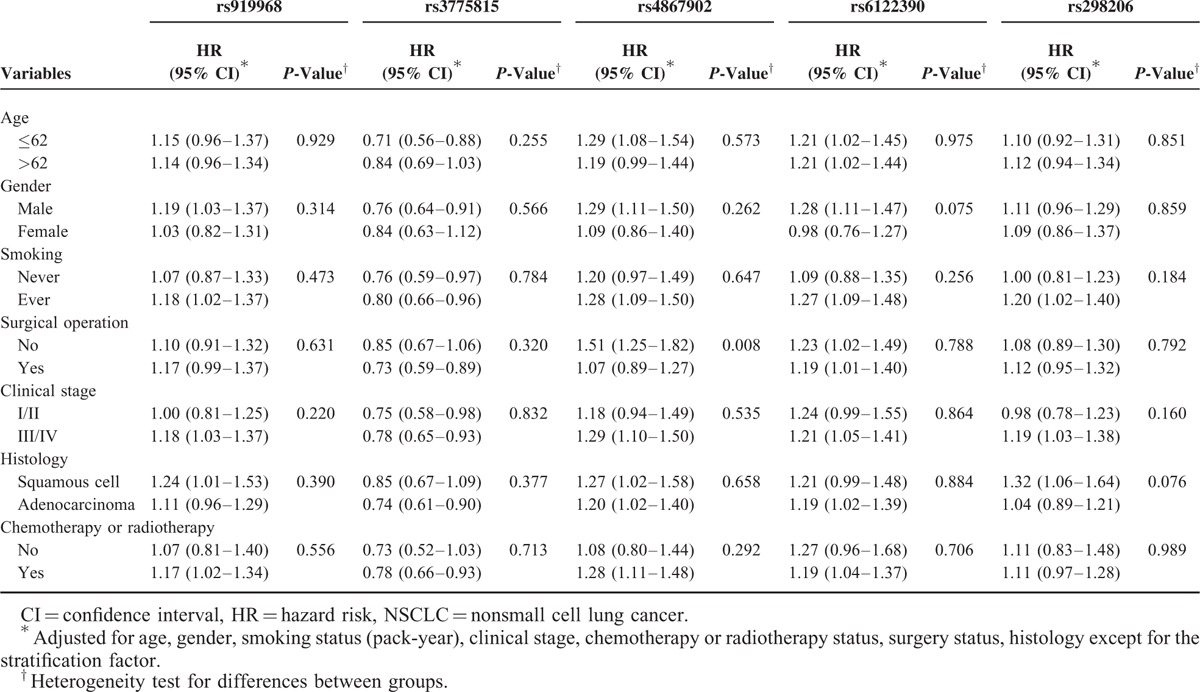
Stratification Analysis of Polymorphism Genotypes Associated With NSCLC Survival

In addition, statistically significant multiplicative interactions were discovered between the genotypes of rs4867902 and surgical operation status (*P*_int_ = 0.013, Table 5).

**TABLE 5:**
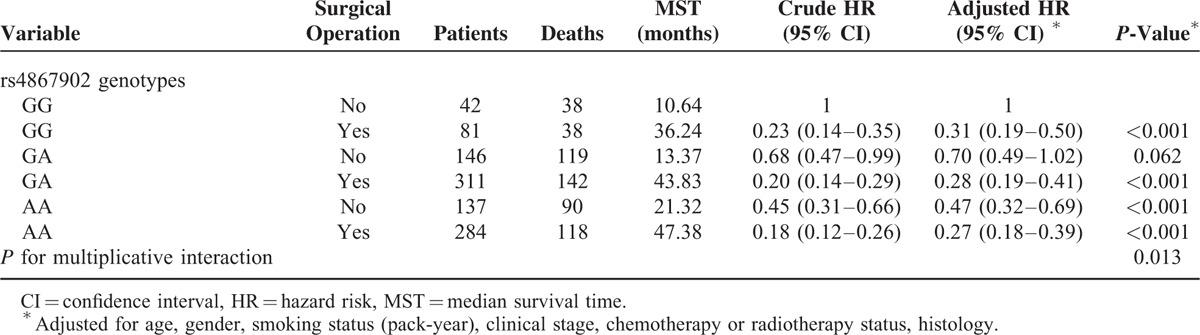
The Interaction Analysis Between rs4867902 and Surgical Operation

## DISCUSSION

In the study, we conducted a clinical follow-up to investigate the potential associations of several genetic variations in *miR-184* and the members of *miR-218* and *miR-124* family with the survival of NSCLC patients. We found that 5 SNPs (*miR-184* rs919968, *miR-218-1* rs3775815, *miR-218-2* rs4867902, *miR-124-2* rs298206, and *miR-124-3* rs6122390) indicated significant associations with the prognosis of NSCLC in a Chinese population. Additionally, the combined analysis of these 5 SNPs indicated a remarkable locus-response effect between number of unfavorable alleles (rs919968-A, rs3775815-A, rs4867902-G, rs6122390-A, and rs298206-T) and the death risk of NSCLC.

Aberrant expressions of miRNAs are closely related to biological and clinical features of specific tumors in human.^22^ Several studies have indicated *miR-184* functions as a potential onco-miRNA in some cancers and plays a role in cell proliferation and apoptosis.^23^ For example, a study showed that *miR-184* functioned as an oncogenic modulator in hepatocellular carcinoma (HCC), and miR-184 might play a part in the proliferation of HCC cells by affecting the expression of inositol polyphosphate phosphatase-like 1 (INPPL1) and act as an anti-apoptotic cytokine in HCC development through suppressing the activities of caspases 3/7.^24^ However, no study has investigated the associations between polymorphisms of *miR-184* and cancer development. In our study, we found that rs919968 variant A was associated with the worse prognosis of NSCLC. rs919968 is located at upstream 4784 bp of *miR-184* and a web-based SNP analysis tool (http://snpinfo.niehs.nih.gov/) indicated that rs919968 might regulate the transcription by intervening the activities of transcription factor binding sites (TFBS) and then influence the expression of *miR-184* and target genes.

*MiR-218* matures from *miR-218-1* and *miR-218-2*, 2 loci of which were located within the introns of *SLIT2* (4p15.31) and *SLIT3* (5q35.1), respectively.^9^ Wu et al^12^ reported that reduced expression of *miR-218* was associated with worse survival of lung cancer. Furthermore, some functional studies showed that, as the expression of *miR-218* increased, cell migration was inhibited and the expression of high mobility group box-1 (*HMGB1*) was also suppressed when *miR-218* targeted its 3′-untranslated region (UTR) in NSCLC.^25^ Some studies have examined the associations of rs11134527 located at putative promoter region of *miR-218* with the risk of different human cancers, such as esophageal squamous cell carcinoma^26^ and cervical cancer.^27^ However, this SNP was excluded in our study because of a low call rate (51.2%) in the HapMap database. In our study, we observed 2 other SNPs (*miR-218-1* rs3775815 and *miR-218-2* rs4867902) were significantly associated with the prognosis of NSCLC. rs3775815 and rs4867902 are located at upstream 228 bp of *miR-218-1* and 4364 bp of *miR-218-2*, respectively. Although functional evidence for these 2 SNPs has been unclear, some information as implemented in SNPinfo indicated that these 2 SNPs might have the influence on the binding of transcription factors. Thus, further in-depth studies are needed to validate our findings and find the causal SNPs that actually contribute to the survival of NSCLC patients.

*MiR-124* is transcribed from three precursor isoforms located on 8p23.1 (*miR-124-1*), 8q12.3 (*miR-124-2*), and 20q13.33 (*miR-124-3*). Previous researches suggested that miR-124 could act as a potential tumor suppressor, and was epigenetically silenced in many types of cancers.^28–30^ Recent researches have further indicated that miR-124 was involved in certain malignant processes including tumor proliferation, metastasis, angiogenesis, and epithelial–mesenchymal transition (EMT) by targeting several important genes in cancers.^13,16,17,29,31^ Additionally, the expression level of miR-124 has been identified to be associated with the prognosis of lung cancer.^17^ However, few studies on associations between polymorphisms of *miR-184* and cancer progress have been reported. In this study, we found that 2 SNPs (*miR-124-2* rs298206 and *miR-124-3* rs6122390) located 9207 bp upstream of *miR-124-2* and 4588 bp of *miR-124-3* respectively, were associated with the worse survival of NSCLC patients. SNPinfo also indicated TFBS of these SNPs, which may be a potential mechanism for the superficial association between 2 SNPs and the poor prognosis of NSCLC patients. However, the data of these 2 SNPs should be interpreted with caution as the associations with NSCLC survival were not robust as assessed by Bonferroni correction.

In conclusion, our findings indicated that several potentially functional SNPs in *miR-184*, *miR-218-1*, *miR-218-2*, *miR-124-2*, and *miR-124-3* were probably novel predictors for NSCLC prognosis in Chinese patients. Large better-designed researches with a variety of populations and as well as functional assessments are in great need to verify and extend our findings.
